# Green-Engineered Clays Tightly Adsorb and Detoxify Environmentally Persistent Polychlorinated Biphenyls and Complex Mixtures

**DOI:** 10.3390/toxics14070573

**Published:** 2026-06-29

**Authors:** Johnson O. Oladele, Xenophon Xenophontos, Phanourios Tamamis, Stephen Safe, Timothy D. Phillips

**Affiliations:** 1 Interdisciplinary Faculty of Toxicology, Texas A&M University, College Station, TX 77843, USA; oladelejohn2007@tamu.edu (J.O.O.); ssafe@cvm.tamu.edu (S.S.); 2 Department of Veterinary Physiology and Pharmacology, College of Veterinary Medicine & Biomedical Sciences, Texas A&M University, College Station, TX 77843, USA; 3 Artie McFerrin Department of Chemical Engineering, College of Engineering, Texas A&M University, College Station, TX 77843, USA; xxenop01@tamu.edu (X.X.); tamamis@tamu.edu (P.T.); 4 Department of Materials Science and Engineering, College of Engineering, Texas A&M University, College Station, TX 77840, USA

**Keywords:** polychlorinated biphenyl, adsorption isotherms, dioxin, clay, organoclays, molecular dynamics

## Abstract

Commonly occurring polychlorinated biphenyls (PCBs) in the environment have been linked to a broad range of adverse toxicological effects in both animals and humans. In this study, in vitro, in silico, and in vivo models were used to investigate the surface interactions of PCBs with green-engineered clays (GECs). Earlier studies showed that these GECs significantly reduced the toxicities of important planar aromatic chemicals such as benzene and aflatoxin B_1_ along with ochratoxin A, a chlorinated aromatic chemical. The overall objective for this study was to show that GECs could tightly adsorb PCBs, resulting in a decrease in toxicity of a commercial PCB mixture (Aroclor 1260). Gastrointestinal pH and temperature were simulated in vitro, and the clay surface binding interactions of six PCBs were characterized using isothermal analyses. Molecular dynamics (MD) simulations were employed to provide atomistic understanding into PCB congener interactions with parent and chlorophyll-amended clays. To confirm the ability of GECs to protect a living organism, Aroclor 1260 was investigated using a well-established hydra bioassay. According to simulations, coplanar PCBs had an increased probability of binding to parent clay compared to non-coplanar ones, in line with experiments, due to their ability to lay flat on the clay surface. Chlorophyll amendments enhanced binding of all PCBs according to both experiments and computations. Within the simulations, chlorophyll amendments facilitated both coplanar as well as non-coplanar PCBs to directly bind to the clay and additionally interact with chlorophyll amendments, as well as to bind to chlorophyll amendments without necessarily interacting with the clay. Aroclor 1260 caused irreversible damage to hydra. At 0.05% inclusion, parent clay offered limited protection (20%) while GECs offered 55% to 65% protection, showing the advantage of GECs over parent clays. The findings of this study indicate that edible GECs adsorb PCBs, with the highest sorption associated with the coplanar congeners. Further studies are warranted to determine the application of GECs as potential disaster-response supplements in the diet to reduce the bioavailability of PCBs from contaminated food and water, especially following floods and other emergencies.

## 1. Introduction

Polychlorinated biphenyls (PCBs) are a class of synthetic organic chemicals consisting of 209 congeners which are different in the number and positions of chlorine atoms in their structure [1,2]. PCBs were initially synthesized in 1876 and gained industrial significance throughout the 20th century. They were predominantly used as dielectric fluids in capacitors and transformers, as well as in plasticizers, lubricants, and hydraulic systems. These applications were supported by their physicochemical stability, high thermal resistance, and non-flammability [3,4]. PCBs on a global scale have been designated as persistent organic pollutants (POPs) under the Stockholm Convention in 2001 which reflects their environmental persistence, bioaccumulation, toxicity and potential for long-range transport [5].

The prevalence of polychlorinated biphenyls (PCBs) in the environment has been linked to a broad range of adverse toxicological effects in both animals and humans. These include carcinogenicity, immunosuppression, neurotoxicity, obesity, diabetes, and endocrine disruption. For instance, dioxin-like coplanar congeners (such as PCB77, PCB126) elicit toxic effects primarily via activation of the aryl hydrocarbon receptor (AhR) which leads to dysregulation of gene expression involved in immune function, xenobiotic metabolism, and development. Similarly, recent reports showed that both coplanar and non-coplanar PCBs contribute to oxidative stress, mitochondrial dysfunction, and endocrine disruption across multiple species [6,7].

The toxic effects of PCBs established a compelling need for effective remediation strategies. High volumes of legacy waste and environmental sustainability standards are two problems that existing mitigation strategies frequently fail to address. Traditional thermal techniques (e.g., thermal desorption and incineration) require a lot of energy and are difficult to operate. They often lead to the formation and release of toxic secondary volatile compounds into the ecosystem. Incorporating naturally derived materials to stabilize PCBs offers a more economical, scalable, and sustainable solution. Clay minerals have been shown to be very effective adsorbents of diverse chemicals. They are naturally available, not expensive, and have unique structural and chemical properties that enhance their toxin-binding capacity. Nonetheless, parent clays are inherently hydrophilic, which limits their capacity to sequester highly non-polar, hydrophobic organic pollutants such as PCBs. Therefore, to improve their adsorption capacity, smectite clays are frequently functionalized through modification by surfactants within their interlayers and on basal surfaces to produce lipophilic organoclays. This process improves their functionality, selectivity, surface properties and overall adsorption performance [8,9].

This study examined the activity and adsorption capacity of green-engineered clays (GECs) to bind six PCBs (coplanar and non-coplanar congeners) in water. These congeners include coplanar PCB77 (3,3′,4,4′-tetrachlorobiphenyl) and PCB126 (3,3′,4,4′,5-pentachlorobiphenyl) (no ortho chlorine atoms) [10] and non-coplanar PCB157 (2,3,3′,4,4′,5′-hexachlorobiphenyl), PCB153 (2,2′,4,4′,5,5′-hexachlorobiphenyl), PCB154 (2,2′,4,4′,5,6′-hexachlorobiphenyl), and PCB155 (2,2′,4,4′,6,6′-hexachlorobiphenyl), (with one, two, three, and four ortho chlorine atoms, respectively) containing dihedral angles of 57° and 74° [11,12]. The major difference in these PCBs is the rotation about the single carbon–carbon bond connecting the two phenyl rings [13]. Also, the chlorine atoms in PCB molecules induce changes to the conformational behavior of the biphenyl moiety, with the position and number of chlorine atoms dictating the extent and nature of these changes [1,10,14]. Chlorine atoms at ortho positions exert the most profound effect, due to their large size, sterically hindering free rotation around the single carbon–carbon bond connecting the two phenyl rings [13,14]. Multiple ortho chlorine atoms can effectively “lock” the molecule into non-coplanar conformations, while the lack of ortho chlorine atoms (or the presence of just one) enables the molecule to adopt nearly planar geometries [1,13].

PCB binding and interactions were measured using the well-established methods of isothermal analyses and isothermal adsorption modeling, while molecular dynamics (MD) simulations, along with other computational approaches, were used to explore with atomistic detail of the binding properties of PCBs to parent clays and GECs. To confirm the capability of the GECs to protect a living organism, a commercial mixture of PCBs, Aroclor 1260, was investigated using a well-established hydra assay.

## 2. Materials and Methods

### 2.1. Materials and Chemicals

Chlorophyll and chlorophyllin were obtained from Santa Cruz Biotechnology (Dallas, TX, USA) and Aldrich Chemical Co. (Milwaukee, WI, USA), respectively. Calcium montmorillonite (CM) with an external surface area of ~70 m^2^ g^−1^, total surface area of ~850 m^2^ g^−1^, and cation exchange capacity of 97 cmol kg^−1^ was acquired from BASF (Lampertheim, Germany). Sodium montmorillonite (SM) from Halliburton (Houston, TX, USA) was extracted from mines in Wyoming with an estimated cation exchange capacity (CEC) equal to 75 cmol kg^−1^. The generic formula for these clays is (Na,Ca)_0.3_(Al,Mg)_2_Si_4_O_10_(OH)_2_·nH_2_O [15]. Ultrapure deionized water (18.2 MΩ) was obtained from an Elga™ automated filtration system (Woodridge, IL, USA). All PCBs and Aroclor 1260 were gifts from Dr Safe’s laboratory at Texas A&M University [16,17]. pH calibration buffers (4.0, 7.0, and 10.0) and high-pressure liquid chromatography (HPLC)-grade acetonitrile were obtained from VWR (Atlanta, GA, USA).

### 2.2. Synthesis and Characterization of GECs

GECs were prepared through the modification of the parent smectite clays (SM and CM) following the previously developed protocols [8,18,19]. This procedure involved amending the clays at 150% CEC to ensure effective saturation of exchangeable sites with chlorophyll (SMCH and CMCH) and chlorophyllin (SMCHIN and CMCHIN). To enhance maximal surface functionalization and intercalation, CM or SM was suspended at 5% (*w*/*w*) in acidified ultrapure deionized water (pH 4.2) and rapidly mixed with either chlorophyll or chlorophyllin for 24 h. After this, the clay suspensions were centrifuged at 3000× *g* for 20 min, and the resulting GECs were washed repeatedly with ultrapure deionized water to remove unbound chlorophyll or chlorophyllin. After three successive wash cycles, the GECs were dried under desiccation, homogenized and sieved to obtain a uniform particle size (<150 µm).

The structural stability and integrity of chlorophyll and chlorophyllin within the clay interlays, as well as a comprehensive physiochemical characterization of the GECs, has been reported in our previous studies [18,19]. Key physiochemical properties relevant to environmental performance including pH, hydrophobicity, water expansibility coefficient, moisture content, bulk density and point of zero charge (pH_pzc_) have been reported [18,19]. The lipophilicity, light sensitivity, expansibility, particle size distribution, and surface charge (zeta potential) as well as detailed morphological and structural analysis using scanning electron microscopy (SEM), X-ray powder diffraction (XRD) and Fourier-transform infrared spectroscopy (FTIR) have been reported [18,20].

### 2.3. Adsorption Isothermal Analysis

The adsorption isothermal experiments were carried out by following established methods [8,12,15]. Briefly, PCB stock solutions were prepared by dissolving the crystalline congeners in acetonitrile. Working solutions of 15 ppm of each PCB were prepared by dissolving appropriate amounts of the stock solution into ultrapure deionized water adjusted to pH 2 or 6. Each GEC and parent clay was introduced as a 2 mg/mL dispersion into the series of PCB solutions in a defined concentration gradient ranging from 10% to 100%. Three treatment controls which included PCB solution without clay, clay solution alone, and ultrapure deionized water as blank control were used in this study. All experimental and control tubes were covered and agitated at 1000 rpm for 2 h at 37 °C. Thereafter, samples were centrifuged at 2000× *g* for 5 min to separate the clay–PCB complex from the unbound PCB in the supernatant.

The PCB concentrations in the supernatants were quantified using UV–visible scanning spectrophotometry, a technique that has been widely used for Aroclor mixtures and PCB congeners [12,21,22]. The detection wavelengths for PCB 157, PCB 155, PCB 154, PCB 153, PCB 126 and PCB 77 were 254.9 nm, 236.7 nm, 245.8 nm, 207.2 nm, 264.5 nm, and 260.9 nm, respectively.

### 2.4. Data Analysis, Calculations and Curve Fitting

The concentrations of unbound PCBs (PCB157, PCB155, PCB154, PCB153, PCB126 and PCB77) in the solution were determined using UV–visible scanning spectrophotometry. The quantity of PCBs adsorbed onto the sorbent materials was obtained by subtracting the concentrations in the test samples from the values observed in the corresponding PCB controls. Adsorption data were standardized as mol/kg and depicted in isotherm plots. Curve fitting and data analysis were conducted using Table-curve 2D V. 5.01 (Systat Software) and the R programming language enabling evaluation of the adsorption parameters [23,24]. Non-linear regression analysis was performed to derive model parameters, and the selection of the best-fit adsorption model was guided by the correlation coefficients (r^2^) and residual distribution from triplicate studies. Established adsorption isotherms, specifically Langmuir and Freundlich [15], were deployed to interpret the experimental results.

The Freundlich isotherm, suitable for describing adsorption on a heterogeneous surface, is represented by

(1) $$q=K_{f}\;C_{w}^{1/n}$$

where K_f_ is the Freundlich constant and 1/n indicates surface heterogenicity.

For the Langmuir model, adsorption on a homogeneous surface was characterized using

(2) $$q=Q_{\max}\frac{K_{d}C_{w}}{1+K_{d}C_{w}}$$

where *Q*_max_ = maximum binding capacity (mol kg^−1^), *K*_d_ is the Langmuir distribution constant, *C*_w_ is the equilibrium concentration of PCBs (mol L^−1^), and *q* = the amount of PCBs adsorbed (mol kg^−1^).

The standard Gibbs free energy change (Δ*G*°) of the adsorption was calculated using the Gibbs free energy equations:

ΔG = ΔG° + RTInKe°(3)

To obtain Ke° from K_d_, the following equation was used:

Ke° = K_d_ × [C°] (4)

where Ke° is the dimensionless equilibrium constant, C° is standard concentration = 1 mol/L, *T* (absolute temperature) is 273 + *t* (°C), and *R* (gas constant) is 8.314 J/(mol K).

Adsorption was considered spontaneous when Δ*G*° < 0; when Δ*G*° > 0, the adsorption process was considered not energetically favorable and at equilibrium, Δ*G*° = 0.

### 2.5. Computational Studies and Molecular Simulation

The binding properties of six different PCB molecules (PCB77, PCB126, PCB153, PCB154, PCB155, and PCB157) to parent clay (in the absence of amendments; CM) and to chlorophyll-amended clay (CMCH) were investigated, individually for each, using MD simulations. Simulations were performed examining acidic as well as near-neutral conditions, in conjunction with experiments. The PCB model structures were extracted from PubChem [25] (PCB77: 36187, PCB126: 63090, PCB153: 37034, PCB154: 63082, PCB155: 36647, and PCB157: 50891), while the chlorophyll (Chlorophyll-A) model structure was taken from PDB [26,27]. These model structures were utilized as initial structures within the simulations corresponding to both acidic and near-neutral conditions. The initial structure for CM in acidic conditions was generated with CHARMM-GUI Nanomaterial Modeler [28,29,30,31], based on INTERFACE FF [32] as of November 2024 [33,34], followed by specific adjustments and modifications described in our previous studies [18,19,33,34,35,36]. The initial CM structure in near-neutral conditions was constructed following how we modeled neutral clay in previous studies [34,35], using the initial structure of CM in acidic conditions and removing specific hydrogen atoms and hydroxyl groups from the clay edges, based on differences between the acidic and neutral calcium montmorillonite models of INTERFACE FF [32]. Montmorillonite clay (in both pH conditions) was parameterized using INTERFACE FF [32] in conjunction with CHARMM-GUI [28,29,30,31]. PCB molecules were parameterized using CGenFF [37], while for chlorophyll, we used a combination of parameters derived from CGenFF along with parameters from [38], as described in our previous studies [18,19,33,36].

To amend clay with chlorophyll molecules, a single simulation of 100 ns was performed in acidic conditions. Initially, clay was centered in an 85 × 85 × 85 Å^3^ cubic water box and 12 copies of chlorophyll were placed randomly in the box such that any atom of each chlorophyll molecule would be at least 5 Å away from any other chlorophyll molecule or clay layers. Neutralizing calcium ions were randomly introduced within the water box. An equilibration stage at constant volume was performed for 200 ps at 300 K, before the production stage at 300 K and 1 atm (using an isotropic barostat) for 100 ns with magnesium/aluminum atoms of CM being constrained, in conjunction with our previous studies [18,19,33,34,35,36]. The final simulation snapshot was extracted and was utilized as the initial structure of CMCH in subsequent simulations investigating PCBs binding properties in acidic conditions. The initial structure of CMCH in near-neutral conditions was constructed using the initial structure of CMCH in acidic conditions following how we modeled CM in near-neutral conditions, while also preserving the coordinates of chlorophyll molecules from the extracted structure.

The binding properties of the six PCB molecules to CM (control) and CMCH were investigated using five independent MD simulation runs for each PCB molecule in conjunction with CM or CMCH in each pH condition, resulting in a total of 120 simulations. Initially, in both acidic and near-neutral conditions, the CM or CMCH initial structure (in the corresponding pH condition) was centered in a 130 × 130 × 130 Å^3^ cubic water box. Four copies of PCB molecules were placed randomly in the box such that any atom of each PCB molecule would be at least 30 Å away from any other PCB or chlorophyll molecule or clay layers. Neutralizing calcium ions were randomly introduced within the water box. An equilibration stage at constant volume was performed for 200 ps at 300 K, before the production stage at 300 K and 1 atm (using an isotropic barostat) for 100 ns with the magnesium/aluminum atoms of the clay being constrained, analogously to our previous studies [18,19,33,34,35,36]. CHARMM-GUI inputs [28,29,30,31] for system and simulation setup as generated in November 2024 for previous studies [33,34] were applied following adjustments and modifications mentioned above. Analysis of the simulations was performed with in-house FORTRAN programs, according to which an interaction (contact) between two entities occurred if any pair of atoms of the two entities was within a cutoff 3.5 Å distance. First, we calculated the binding percentage probabilities of PCBs to CM as well as CMCH, analogously to previous work by us [18,19,33,34,35,36]. To determine the binding mechanisms through which PCBs bind to chlorophyll-amended clay, the interactions were decomposed into direct (binding to clay only), direct-assisted (binding simultaneously to clay and chlorophyll molecules or aggregates bound to clay), and indirect-assisted interactions (binding only to chlorophyll molecules or aggregates bound to clay), in accordance with our previous studies [18,19,33,34,35,36]. Indirect-assisted interactions were further decomposed into two cases: indirect-assisted interactions in the interlayer (where the chlorophyll molecules or aggregates were within the interlayer), and indirect-assisted interactions at the outer surfaces (where the chlorophyll molecules or aggregates were at either the top or bottom outer surface). Additionally, chlorophyll was decomposed into two groups, comprising the chlorin ring (which is referred to as the “head”) as well as the alkyl chain (which is referred to as the “tail”), for the investigation of the interactions between PCB molecules and CMCH. Furthermore, the percentage of atoms per PCB molecule interacting with CM or CMCH (contribution of PCB atoms to binding) as well as the dihedral angle of the PCB molecules were analyzed. The contribution of PCB atoms to binding in directed-assisted interactions was estimated by counting how many atoms of each PCB molecule were in contact with clay or chlorophyll molecules and then dividing by the total number of atoms in the given PCB molecule. Similarly, the corresponding calculation in indirect-assisted interactions considered PCB atoms that were in contact with chlorophyll molecules or aggregates only. All analysis was conducted using the last 50 ns of all simulation trajectories, and errors correspond to standard deviations of the averages over the five simulation trajectories per system. VMD [39] was employed for visualization of the simulation trajectories and for the extraction of snapshots.

### 2.6. Ecotoxicological Bioassay (Hydra vulgaris)

An established *Hydra vulgaris* bioassay for environmental chemicals including PCBs [12] was used to assess the detoxification efficacy of GECs against PCBs exposure. *Hydra vulgaris*, sourced from Environmental Canada (Montreal) were maintained at 18 °C under controlled laboratory conditions. Toxicity was quantified using a morphological scoring system ranging from 1 to 10 [40,41], reflecting the extent of adverse effects following exposure to Aroclor 1260. *Hydra vulgaris* were subjected to 30 ppm of Aroclor 1260 and treated with GECs at 0.05%, 0.1% and 0.2% inclusion rates. Each experiment was performed in independent triplicate. Morphological scoring was done at intervals of 0, 4, 20, 28, 44, 68, and 92 h to track the development of Aroclor 1260-induced toxicity and to assess the protective efficacy of each sorbent materials.

### 2.7. Statistical Analysis

The in vivo experiment including control was carried out in three independent replicates. Data analysis was carried out using a one-way ANOVA followed by Tukey’s post hoc test to assess differences between treatment groups. Results with *p* < 0.05 were considered statistically significant.

## 3. Results and Discussion

### 3.1. Adsorption Isotherms for Coplanar PCB Congeners

**Figure 1:**
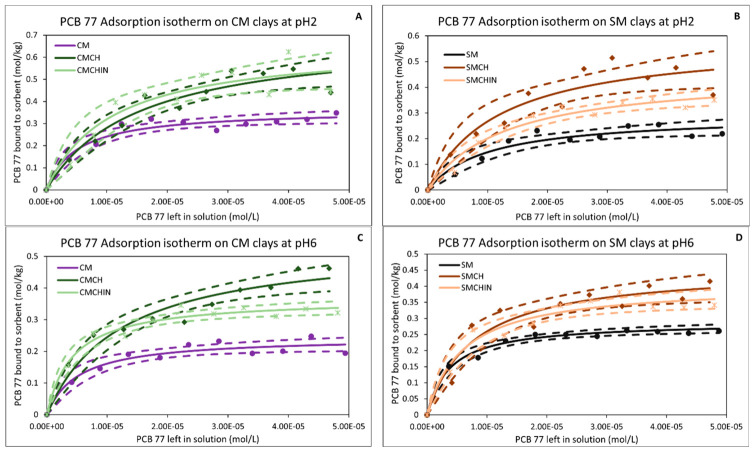
Isotherms showing adsorption of PCB 77 onto binding surfaces of clays at pH 2 (**A**,**B**) and pH 6 (**C**,**D**). CMCH: Chlorophyll-amended calcium montmorillonite; CMCHIN: chlorophyllin-amended calcium montmorillonite; CM: calcium montmorillonite; SMCH: chlorophyll-amended sodium montmorillonite; SMCHIN: chlorophyllin-amended sodium montmorillonite; SM: sodium montmorillonite. The solid lines represent the adsorption isotherm plots based on the Langmuir model, while the dashed lines represent the 95% confidence band of Langmuir model.

**Figure 2:**
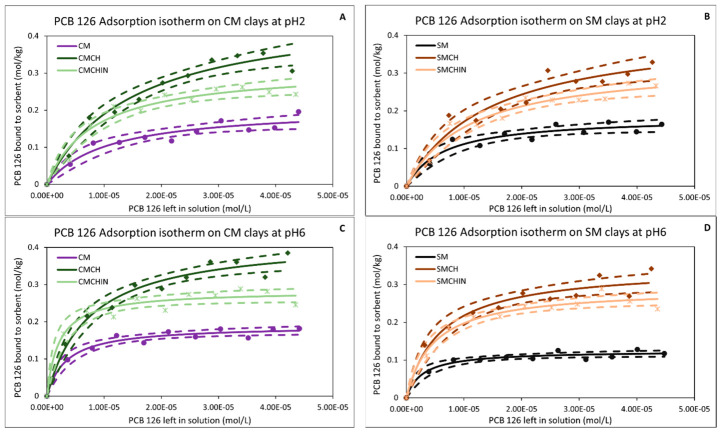
Isotherms showing adsorption of PCB 126 onto binding surfaces of clays at pH 2 (**A**,**B**) and pH 6 (**C**,**D**). CMCH: Chlorophyll-amended calcium montmorillonite; CMCHIN: chlorophyllin-amended calcium montmorillonite; CM: calcium montmorillonite; SMCH: chlorophyll-amended sodium montmorillonite; SMCHIN: chlorophyllin-amended sodium montmorillonite; SM: sodium montmorillonite. The solid lines represent the adsorption isotherm plots based on the Langmuir model, while the dashed lines represent the 95% confidence band of Langmuir model.

The adsorption isotherms of PCB77 and PCB126 bound to the surfaces of parent clays (CM and SM) and the GECs (CMCH, CMCHIN, SMCH and SMCHIN) were evaluated at pH 2 and pH 6 (Figure 1 and Figure 2). All isotherms were fitted to the Langmuir model with correlation coefficients (r^2^) ranging from 0.91 to 0.98 (Table 1). PCB77 and PCB126 are dioxin-like PCBs and have WHO Toxic Equivalency Factors (TEFs) because they activate AhR. Despite frequently being present at low amounts, PCB 126 dominates TEQ, making it particularly significant in risk evaluation. The good fit of all isotherms for the PCB congeners (r^2^ ≥ 0.91) indicated that the GEC surfaces exhibited relatively uniform sorption sites after chlorophyll and chlorophyllin modification. This is consistent with earlier research which demonstrated that chlorophyll and chlorophyllin acted as surfactants that intercalated with the homogenized interlayer hydrophobic domains, thus enhancing monolayer uptake of non-polar organic contaminants such as ochratoxin, aflatoxin, benzene, glyphosate and aminomethylphosphonic acid [20].

At pH 2, both CM and SM showed moderate adsorption capacity (0.36 mol kg^−1^ and 0.29 mol kg^−1^ respectively) for PCB77 (Figure 1A,B) and values of 0.21 mol kg^−1^ and 0.18 mol kg^−1^, respectively, for PCB126 (Figure 2A,B). GECs exhibited 1.5–2.0-fold higher adsorption capacities compared to the parent clays (Table 1). This indicated an enhancement of the binding capacity of the clays by chlorophyll and chlorophyllin. Chlorophyll clays (CMCH and SMCH) demonstrated higher adsorption capacity ranging from 0.60 to 0.70 mol kg^−1^ for PCB77 and 0.42 to 0.47 mol kg^−1^ for PCB126. Similarly, chlorophyllin clays (CMCHIN and SMCHIN) showed good adsorption capacity ranging from 0.46 to 0.61 mol kg^−1^ for PCB77 and 0.31 to 0.33 mol kg^−1^ for PCB126. The K_d_ magnitudes ranged from 10^4^ to 10^5^ suggesting strong sorption affinity across the clays. The ∆G° values for parent clays and the GECs ranged from −21.70 ± 1.19 to −23.60 ± 1.15 kJ/mol indicating that the adsorption of PCB77 and PCB126 was spontaneous and in the forward direction.

At pH 6, there was a slight decrease in the adsorption capacity of the parent clays and GECs. However, the latter maintained higher capacity than the parent clays. The K_d_ values (10^4–5^) showed strong sorption affinity and negative ∆G values (−21.30 ± 1.50 to −23.70 ± 1.30 kJ/mol) indicating that adsorption was spontaneous. The steady improvement of Q_max_ in GECs demonstrates the vital role of organic functional groups (long phytol tail in chlorophyll) in facilitating hydrophobic interactions. Previous studies have shown that chlorophyll increases the basal spacing and created non-polar regions [8] that favor portioning and forming tight complexes with planar aromatics (such as aflatoxin B1) [33] and chlorinated aromatics (such as ochratoxin A) [19]. This is consistent with MD simulations. The similarity in the adsorption patterns between CM- and SM-derived GECs indicates that the type of parent clay (calcium or sodium montmorillonite) is less significant than the nature of the organic modification. Both show sorption for some congeners consistent with their swelling characteristics and cation exchange capacity.

### 3.2. Adsorption Isotherms for Non-Coplanar PCB Congeners

**Figure 3:**
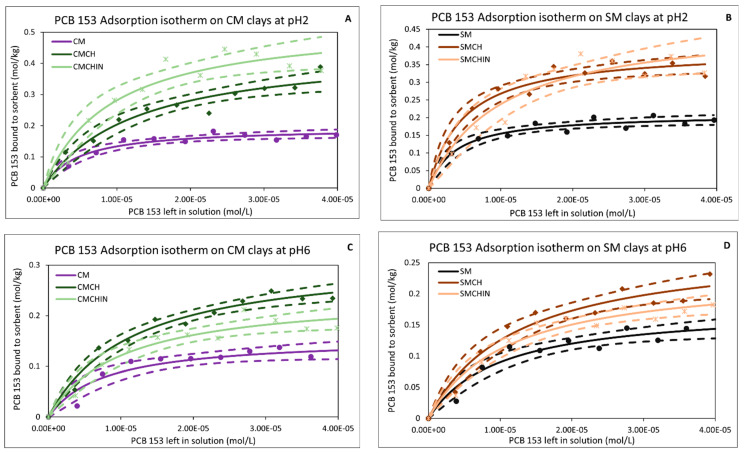
Isotherms showing adsorption of PCB 153 onto binding surfaces of clays at pH 2 (**A**,**B**) and pH 6 (**C**,**D**). CMCH: Chlorophyll-amended calcium montmorillonite; CMCHIN: chlorophyllin-amended calcium montmorillonite; CM: calcium montmorillonite; SMCH: chlorophyll-amended sodium montmorillonite; SMCHIN: chlorophyllin-amended sodium montmorillonite; SM: sodium montmorillonite. The solid lines represent the adsorption isotherm plots based on the Langmuir model, while the dashed lines represent the 95% confidence band of Langmuir model.

Adsorption of the four non-coplanar PCB congeners (PCB153, PCB154, PCB155 and PCB157) bound to the surfaces of parent clays (CM and SM) and the GECs (CMCH, CMCHIN, SMCH and SMCHIN) was evaluated at pH 2 and pH 6 (Figure 3 and Figures S1–S3). Across all tested systems, the equilibrium data fit well with the Langmuir model with correlation coefficients (r^2^) ranging from 0.91 to 0.97 (Table 2). This indicated monolayer adsorption on relatively homogeneous binding domains of the parent clay and those created by the green modification processes using chlorophyll and chlorophyllin in the GECs.

At pH 2, the Q_max_ values varied significantly between the parent clays and the GECs, revealing structural differences imparted by chlorophyll and chlorophyllin functionalization. For instance, Q_max_ for PCB153 ranged from 0.19 mol kg^−1^ (CM) to 0.45 mol kg^−1^ (CMCH) and 0.54 mol kg^−1^ (CMCHIN). Q_max_ ranged from 0.21 mol kg^−1^ (SM) to 0.39 mol kg^−1^ (SMCH) and 0.47 mol kg^−1^ (SMCHIN). Similar patterns were noted for PCB154 where GECs increased the Q_max_ from 0.24 mol kg^−1^ (CM) and 0.18 mol kg^−1^ (SM) to 0.37–0.38 mol kg^−1^ and 0.34–0.38 mol kg^−1^, respectively. Interestingly, both GECs showed almost the same Q_max_ for PCB 157. Across all congeners, the parent clays (CM and SM) exhibited similar capacities while the chlorophyll (-CH) and chlorophyllin (-CHIN) GECs demonstrated 1.5–2.0-fold higher adsorption capacities compared to the parent clays.

At pH 6, adsorption behavior followed similar trends but with a slight reduction in binding capacities. The observed slight reduction in binding of GECs for all non-coplanar PCB congeners is consistent with weaker electrostatic contributions under near-neutral conditions. The K_d_ magnitudes ranged from 10^4^ to 10^6^, indicating strong sorption affinity across the clay matrix. PCB153 displayed the highest affinity with K_d_ values up to 2.19 × 10^6^ for CM at pH 2. The free energy values (∆G) ranged from −19.40 ± 1.05 to −22.40 ± 0.98 kJ mol^−1^, confirming spontaneous adsorption in the forward direction. The values of ∆G indicate that adsorption is dominated by strong physical interactions, primarily hydrophobic and van der Waal forces rather than chemisorption.

There was a significant decrease in sorption efficacy in non-coplanar congeners compared to the coplanar congeners. This could be accounted for by steric effects: the positioning of the two phenyl rings varies chemically with the degree of ortho substitution. For congeners lacking ortho chlorine atoms (PCB126 and PCB77), the biphenyl rings adopt a dihedral angle of about 44°. When ortho substitution occurs on only one ring as in PCB157, the dihedral angle increases to 57°. However, congeners carrying ortho substitutions on both phenyl rings (PCB153, PCB154 and PCB155) exhibit a remarkably more twisted configuration with dihedral angles equal to 74° [11]. This result supports earlier studies where coplanar PCBs exhibited higher sorption than non-coplanar congeners [42].

The maximum adsorption capacity (Q_max_), which showed increases in orders of magnitude in chlorophyll-GECs in comparison to the chlorophyllin-GECs and native smectite, is an important empirical finding. For sorption, unmodified smectite clays usually rely on small, high-energy surface sites or constrained, hydrated interlayer regions, which frequently result in a reduced binding capacity for hydrophobic organic contaminants including PCBs. All of the six PCB congeners have higher Q_max_ values in the GECs, indicating that bulk hydrophobic partitioning has replaced simple surface adsorption as the underlying sorption mechanism. Several important structural changes are accomplished by the chlorophyll and chlorophyllin intercalation. First, it opens the layered silicate structure by displacing the inorganic exchangeable cations Na^+^ or Ca^+^ through solution intercalation. A quantifiable increase in the basal d_001_-spacing, a necessary structural modification for reaching high capacity, serves as proof of this. Second, the total organic carbon content of the clay is greatly increased by the addition of chlorophyll molecules, which have long, non-polar alkyl chains. As a result, the interlayer space develops a low-density, lipophilic organic domain. Similar to solvent extraction, PCB molecules preferentially partition (dissolve) into this bulk organic phase due to their extremely low aqueous solubility. The overall sorption capacity is maximized by this partitioning into the low-density, loose-packing organic phase, particularly at high contaminant concentrations.

The distribution coefficient (K_d_), a measure of affinity, is high for both coplanar and non-coplanar PCBs. The coplanar, dioxin-like PCBs (PCB77 and PCB126) can interact and access geometrically restricted sites in a tight manner. On the other hand, the non-coplanar congeners (PCBs 153, PCB154, PCB155 and PCB157) are twisted, which results in significant steric hindrance that prevents these molecules from packing optimally or from accessing confined sites. The scenario indicated that the binding enhancement is bimodal: bulky non-coplanar PCBs benefit from the overall capacity (Q_max_), which is driven by general hydrophobic partitioning, while the coplanar congeners benefit from the enhanced affinity (K_d_), which is driven by a secondary interaction like π-π stacking, which provides a greater energetic reduction. Moreover, the Langmuir model is often fitted by the isotherms for coplanar PCBs, indicating particular, saturable binding sites.

### 3.3. MD Simulations

**Figure 4:**
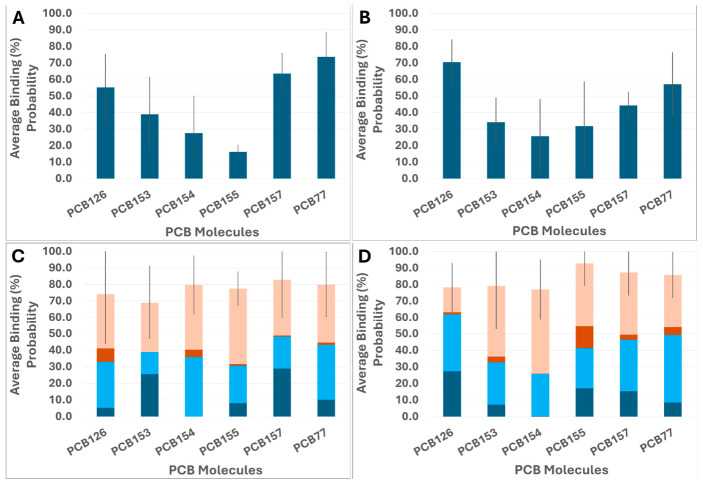
The average percentage binding probabilities of PCB molecules to parent clay (CM) in acidic (**A**) and near-neutral conditions (**B**), and to chlorophyll-amended clay (CMCH) in acidic (**C**) and near-neutral conditions (**D**). Direct interactions, direct-assisted interactions, indirect-assisted interactions in the interlayer, and indirect-assisted interactions at the outer surfaces are shown in dark blue, cyan, dark orange, and light orange, respectively. The average values were calculated using the last 50 ns of five independent simulations; error bars denote the standard deviation of total binding across simulations.

**Figure 5:**
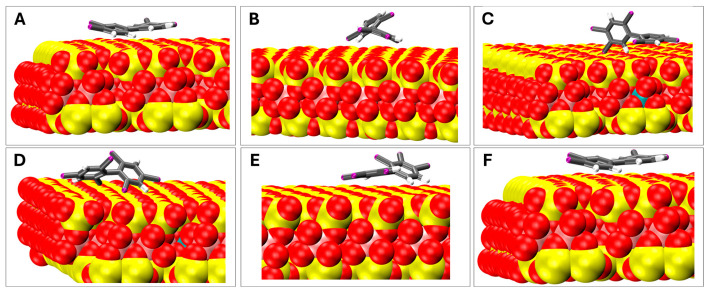
Representative zoomed-in snapshots extracted from MD simulations of PCB126 (**A**), PCB153 (**B**), PCB154 (**C**), PCB155 (**D**), PCB157 (**E**), and PCB77 (**F**), in complex with CM in near-neutral conditions. The clay layers are shown in vdW representation, colored by atom type (Si—yellow; O—red; Mg—pink; Al—Blue-green). The PCB molecules are shown in licorice representation, with the carbon atoms colored in black, the chlorine atoms colored in purple, and the hydrogen atoms colored in white. The hydrogen atoms of the clay, as well as the water molecules and ions, were omitted for clarity. All representations were produced using VMD.

Figure 4A,B show the average binding percentage probability of each PCB to bind CM, in acidic and near-neutral conditions, respectively. Figure 5A–F show the predominant binding modes of PCB126, PCB153, PCB154, PCB155, PCB157, and PCB77, respectively, to CM in near-neutral conditions. The corresponding binding modes to CM in acidic conditions are shown in Figure S4. In both acidic and near-neutral conditions, PCB77, PCB126 and PCB157 had higher overall binding probabilities than PCB153, PCB154 and PCB155 (Figure 4A,B). This could be attributed to their capacity to predominantly adopt coplanar configurations (PCB77, PCB126) or partly coplanar configurations (PCB157), as shown in Figure 5A, Figure 5F, and Figure 5E, respectively. Coplanar configurations correlated with higher percentage contribution of PCB atoms to binding, in both acidic and near-neutral conditions, as shown in Figures S5 and S6, respectively. On the contrary, PCB153, PCB154 and PCB155 could not adopt coplanar configurations and lay flat on the clay, as shown in Figure 5B, Figure 5C and Figure 5D, respectively. This is also demonstrated by their dihedral angles in combination with a lower percentage contribution of PCB atoms binding to clay, in both acidic and near-neutral conditions, as shown in Figures S5 and S6, respectively.

The overall trend of the computationally calculated binding probability of each PCB to bind to CM (Figure 4A,B) is in accordance with the experimentally determined binding capacities (Table 1 and Table 2). The slightly higher binding capacity of PCBs observed experimentally in acidic conditions compared to near-neutral conditions could be attributed to differences in the clay edge sites, associated with a ~10% higher probability of hydrogen bonds between PCB chlorine atoms and clay edge atoms within the simulations. This increase conforms with the slightly higher binding capacity of PCB157, PCB77 and to a lesser extent PCB153 and PCB154 in acidic compared to near-neutral conditions within the simulations. This trend was not observed for PCB126, for which the experimental binding capacity values in both conditions were similar, while the trend was also not observed for PCB155 for which the binding capacity according to simulations was the lowest. Nonetheless, it is also important to note the overall high standard deviation values of the computations, showing some level of variability across simulated replicates for all PCBs. The high standard deviation values could be attributed to the small number of PCB molecular copies per system (equal to 4), aimed to mimic to some extent the experimental conditions, as well as to avoid a high degree of aggregation between PCB molecules observed in higher concentrations.

**Figure 6:**
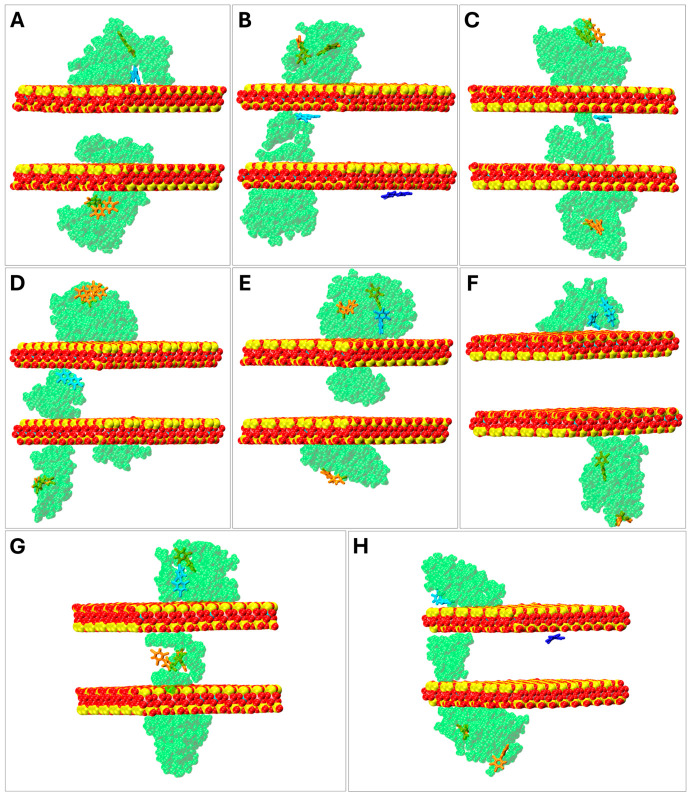
Representative snapshots extracted from MD simulations of PCB126 (**A**,**B**), PCB77 (**C**,**D**), PCB153 (**E**), PCB154 (**F**), PCB155 (**G**), and PCB157 (**H**), in complex with CMCH in near-neutral conditions. The clay layers are shown in vdW representation, colored by atom type (Si—yellow; O—red; Mg—pink; Al—Blue-green). Chlorophyll molecules are shown in transparent vdW representation, colored in green. The PCB molecules are shown in licorice representation, colored in blue, cyan or orange, corresponding to direct, direct-assisted, and indirect-assisted interactions, respectively. The hydrogen atoms of clay, as well as water molecules and ions were omitted for clarity. All representations were produced using VMD.

CMCH enhanced the binding of all PCB molecules investigated in both acidic as well as near-neutral conditions (Figure 4C and Figure 4D, respectively). Interestingly, even though only particular PCB molecules could adopt (PCB126 and PCB77) or could partly adopt (PCB157) coplanar configurations and could directly bind to clay surfaces with high probability, all PCB molecules, irrespective of their geometry, showed the capacity to form direct-assisted interactions (i.e., PCBs interact simultaneously both with clay and bound chlorophyll molecules or aggregates). This prompted us to elucidate how either coplanar or non-coplanar PCBs can contact the clay via direct-assisted interactions. In most cases of direct-assisted interactions, both coplanar (PCB126, PCB77, and partly PCB157) and non-coplanar PCB congeners (PCB153, PCB154, and PCB155) predominantly interacted with chlorophyll, while they maintained a few contacts with clay, in both acidic and near-neutral conditions. The percentage atom contribution of PCBs binding to clay of CMCH is lower than the corresponding percentage in CM, especially for PCBs that are coplanar, which have less tendency to lay flat in CMCH compared to CM (Figures S5 and S6 in acidic and near-neutral conditions, respectively). Example cases of PCB126, PCB77, PCB153, PCB154, PCB155, and PCB157 interacting primarily with chlorophyll while maintaining a few contacts with clay are shown in Figure 6A, Figure 6D, Figure 6E, Figure 6F, Figure 6G and Figure 6H, respectively, in cyan licorice representation. In rare cases of direct-assisted interactions, coplanar congeners (PCB126 and PCB77), could lay flat on the clay and interact simultaneously with neighboring chlorophyll molecules, both in acidic and near-neutral conditions. Example cases of PCB126 and PCB77 lying flat on clay while simultaneously interacting with chlorophyll in near-neutral conditions are shown in Figure 6B and Figure 6C, respectively, in cyan licorice representation. Overall, both coplanar and non-coplanar PCB congeners mostly retained their original geometry while interacting with CMCH in both acidic as well as near-neutral conditions, as depicted by the dihedral angles in Figures S5 and S6, respectively. The corresponding direct-assisted binding modes to CMCH in acidic conditions are shown in Figure S7, in cyan licorice representation.

Additionally, within the simulations, chlorophyll amendments enhanced to a high extent PCB binding through indirect-assisted interactions (i.e., PCBs interact with bound chlorophyll molecules or aggregates, and not clay) in both acidic as well as near-neutral conditions (Figure 4C and Figure 4D, respectively). Example cases of indirect-assisted interactions in near-neutral conditions are shown in Figure 6A,B, Figure 6C,D, and Figure 6E–H for PCB126, PCB77, and PCB153, PCB154, PCB155, and PCB157, respectively, in orange licorice representation. The corresponding indirect-assisted binding modes to CMCH in acidic conditions are shown in Figure S7, in orange licorice representation. Comparison to experiments suggests that the computationally calculated enhancement in PCB binding due to chlorophyll appears overestimated. Indirect-assisted binding to the outer surfaces could potentially be amplified in the simulations, as our simulated model system comprises only an interlayer and two outer surfaces, potentially overestimating outer layer effects. In particular, in the outer surfaces we observe large clumps of chlorophylls attracting most not-directly bound PCBs, while in the interlayer we see smaller aggregates or single chlorophyll amendments attracting the minority of not-directly bound PCBs (Figure S7 and Figure 6, for acidic and near-neutral conditions, respectively). Overall, across direct-assisted and indirect-assisted interactions in both acidic as well as near-neutral conditions, PCB molecules interacted mainly with both the head (chlorin ring) and tail (aliphatic) groups of chlorophyll, simultaneously, or only the tail of chlorophyll and to a lesser extent the head of the chlorophyll only (Figure S8).

### 3.4. Toxic Effects of Aroclor 1260 on Hydra vulgaris and the Protective Role of Organoclays

**Figure 7:**
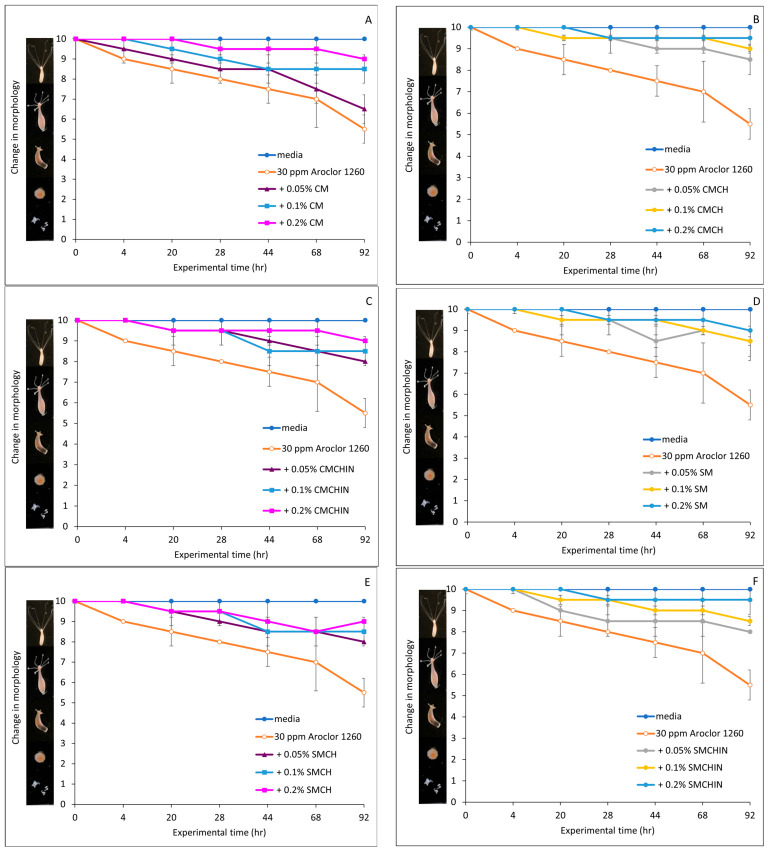
Protective effect of GECs on Aroclor 1260-induced toxicity in *Hydra vulgaris*. (**A**) CM, (**B**) CMCH, (**C**) CMCHIN (**D**) SM, (**E**) SMCH, and (**F**) SMCHIN. CMCH: Chlorophyll-amended calcium montmorillonite; CMCHIN: chlorophyllin-amended calcium montmorillonite; CM: calcium montmorillonite; SMCH: chlorophyll-amended sodium montmorillonite; SMCHIN: chlorophyllin-amended sodium montmorillonite; SM: sodium montmorillonite. The average values were calculated from three independent replicates, and the error bars denote the standard deviation of three independent replicates.

*Hydra vulgaris* are recognized as sensitive organisms for toxin detection and have been employed as an in vivo model to: (a) assess the toxicity of various environmental pollutants including PCBs and Aroclors [12], (b) corroborate findings from in vitro studies, and (c) evaluate the safety and protective capacity of new adsorbent materials against diverse chemical toxicities. Hydra exposed to 30 ppm of Aroclor 1260 in this study demonstrated different toxic responses which led to acute and irreversible morphological damage. As presented in Figure 7, Aroclor 1260 caused 15% morphological damage to the hydra after 20 h of exposure and its toxicity increased to 45% damage after 92 h of exposure which resulted in marked damage to hydra morphology and disintegration of its tentacles which are essential for feeding and locomotion in its aqueous media. This observation was consistent with earlier findings by Wang et al. [12] which established severe damage of *Hydra vulgaris* following exposure to Aroclor 1260.

However, inclusion of the parent clays and GECs showed significant protection at the three inclusion rates (0.05%, 0.1%, and 0.2%) used in this study (Figure 7A–F). The parent clay (CM) exhibited limited protection (20%) at 0.05% inclusion; however, the protection increased as the inclusion rate increased. At the lowest inclusion rate (0.05%), the GECs resulted in 55% to 65% protection. Surprisingly, the GECs resulted in nearly complete protection of hydra at the 0.2% inclusion rate demonstrating a higher GEC sorption affinity for PCB mixtures versus the parent clays. Protection of hydra against Aroclor 1260 toxicity was observed throughout the 92 h exposure duration in this investigation suggesting that GECs could be used as potential toxin binders in aquatic sediments. These results are consistent with previous research on clay-based sorbent strategies for organic pollutants, further supporting their usefulness for mitigating persistent organic contaminants [12,19,36).

## 4. Conclusions

Six polychlorinated biphenyl (PCB) congeners were investigated for their ability to bind onto the surfaces of GECs. The results show that an important modification in the surface properties of common clay minerals resulted in significantly better sorption capabilities than the unmodified clays. The results for both the parent clays and GECs demonstrated that all the PCB congeners fit the Langmuir model, indicating saturable binding sites with high capacities and affinities at both acidic and near-neutral conditions. The GECs had increased capacities for the PCBs in acidic and near-neutral conditions. Moreover, the isotherms of adsorption for all the PCBs were thermodynamically favorable, with ∆G° values ranging from −19.40 ± 1.05 to −22.40 ± 0.98 kJ/mol.

Experiments showed that coplanar PCBs have higher binding capacity for CM compared to non-coplanar PCBs. This finding was in line with independent molecular dynamics (MD) simulations, which depicted that in the case of parent clay, PCB molecules capable of adapting coplanar configurations (PCB77, PCB126, and partly PCB157) were able to align both rings in the same plane, enabling the molecules to bind to clay surfaces and maximizing interactions across both aromatic rings and the clay surface. In contrast, PCB molecules that could not adopt coplanar configurations (PCB153, PCB154, and PCB155), could not optimally bind to clay reflecting a lower binding probability. The ability of particular PCBs to lay flat to CM was in line with observations of previous computational studies [43]. These observations comply with a previous study investigating different PCB molecules, PCB36 and PCB19, coplanar as well as non-coplanar, respectively, showing that coplanar PCBs showed higher adsorption than non-coplanar PCBs [42]. In most cases of direct-assisted interactions, coplanar and non-coplanar PCBs do not lay flat but rather they participate in extensive hydrophobic interactions with chlorophyll molecules. In rarer cases of direct-assisted interactions, and in direct interactions with CM, coplanar PCBs tend to lay flat. Interestingly, these interactions could be facilitated by two aspects: the coplanarity of the molecules as well as the asymmetry of dipoles in their rings (i.e., C-H dipoles oriented from the center of the ring outward and C-Cl dipoles oriented from outwards to the center of the ring according to molecular mechanics parametrization [37]). According to visual inspection, we suggest that the asymmetry could play a role in facilitating PCB126 and PCB77 to lay flat with chlorine atoms tending to orient towards the silicon clay atoms. Coplanarity and partial charge distribution could synergistically play a key role for such direct interactions between PCBs and clay.

The GECs significantly mitigated Aroclor 1260-induced toxicity in *Hydra vulgaris* in the aqueous media. Chlorophyll amendments enhanced binding for all PCBs in experiments and computations. Within simulations, chlorophyll amendments facilitated both coplanar and non-coplanar PCBs to directly bind to the clay and additionally interact with chlorophyll amendments, as well as PCBs to bind to chlorophyll amendments without necessarily interacting with the clay. Our findings highlight the mechanisms through which chlorophyll amendments can enhance PCB binding and can additionally pave the way for the study of chlorophyll amendments as potential effective sorbents for a wider range of environmental contaminants. Taken together, the findings of this study indicate that edible GECs (containing all-GRAS components) adsorb coplanar PCB congeners with the best efficacy. Further studies are warranted to study the application and safety of these “edible” clays as potential supplements in the diet to reduce the bioavailability of PCBs from contaminated food and water, especially following disasters and emergencies.

## Figures and Tables

**Table 1:** Parameters and correlation coefficients from adsorption isotherms for coplanar PCB congeners.

GEC	Adsorption Parameters @ pH 2				Adsorption Parameters @ pH 6			
	Q_max_	K_d_	∆G°	r^2^	Q_max_	K_d_	∆G°	r^2^
**PCB 77**								
CM	0.36	1.99 × 10^5^	−22.90 ± 1.50	0.94	0.24	1.94 × 10^5^	−21.30 ± 1.50	0.93
CMCH	0.70	6.68 × 10^4^	−23.60 ± 1.00	0.93	0.54	8.24 × 10^4^	−23.70 ± 1.30	0.94
CMCHIN	0.61	1.09 × 10^5^	−23.60 ± 1.15	0.92	0.37	2.32 × 10^5^	−22.70 ± 1.59	0.97
SM	0.29	1.02 × 10^5^	−21.70 ± 1.12	0.91	0.29	2.66 × 10^5^	−22.10 ± 1.67	0.98
SMCH	0.60	7.67 × 10^4^	−23.40 ± 1.11	0.94	0.46	1.31 × 10^5^	−23.40 ± 1.29	0.92
SMCHIN	0.46	7.89 × 10^4^	−22.90 ± 0.98	0.96	0.40	1.67 × 10^5^	−22.90 ± 1.44	0.95
**PCB 126**								
CM	0.21	9.56 × 10^4^	−21.70 ± 1.19	0.92	0.19	2.97 × 10^5^	−21.50 ± 1.61	0.96
CMCH	0.47	6.71 × 10^4^	−22.90 ± 0.93	0.96	0.42	1.37 × 10^5^	−23.50 ± 1.36	0.97
CMCHIN	0.31	1.21 × 10^5^	−22.20 ± 1.15	0.96	0.28	4.84 × 10^5^	−22.30 ± 1.84	0.95
SM	0.18	1.56 × 10^5^	−21.20 ± 1.34	0.92	0.12	3.62 × 10^5^	−20.30 ± 1.65	0.95
SMCH	0.42	6.95 × 10^4^	−23.10 ± 1.00	0.94	0.35	1.68 × 10^5^	−23.20 ± 1.48	0.95
SMCHIN	0.33	9.13 × 10^4^	−22.50 ± 1.09	0.96	0.29	2.13 × 10^5^	−22.20 ± 1.49	0.97

∆G°: Gibbs free energy (kJ/mol); K_d_: binding affinity; Q_max_: binding capacity (mol/kg); r^2^: correlation coefficient; CMCH: chlorophyll-amended calcium montmorillonite; CMCHIN: chlorophyllin-amended calcium montmorillonite; CM: calcium montmorillonite; SMCH: chlorophyll-amended sodium montmorillonite; SMCHIN: chlorophyllin-amended sodium montmorillonite; SM: sodium montmorillonite.

**Table 2:** Parameters and correlation coefficients from adsorption isotherms for non-coplanar PCB congeners.

GEC	Adsorption Parameters @ pH 2				Adsorption Parameters @ pH 6			
	Q_max_	K_d_	∆G°	r^2^	Q_max_	K_d_	∆G°	r^2^
**PCB 153**								
CM	0.19	2.19 × 10^6^	−21.60 ± 1.42	0.95	0.16	1.31 × 10^5^	−20.50 ± 1.14	0.90
CMCH	0.45	8.23 × 10^4^	−23.80 ± 1.17	0.95	0.32	8.69 × 10^4^	−22.40 ± 0.98	0.97
CMCHIN	0.54	1.10 × 10^5^	−23.70 ± 1.04	0.93	0.24	1.01 × 10^5^	−21.60 ± 1.02	0.93
SM	0.21	2.76 × 10^5^	−21.90 ± 1.57	0.95	0.18	1.01 × 10^5^	−21.00 ± 1.02	0.94
SMCH	0.39	2.12 × 10^5^	−23.30 ± 1.46	0.96	0.28	8.23 × 10^4^	−22.40 ± 0.94	0.95
SMCHIN	0.47	1.02 × 10^5^	−23.30 ± 1.08	0.91	0.23	9.09 × 10^4^	−21.70 ± 0.95	0.96
**PCB 154**								
CM	0.24	1.14 × 10^5^	−21.80 ± 1.06	0.95	0.14	3.82 × 10^5^	−20.50 ± 1.67	0.92
CMCH	0.37	6.91 × 10^4^	−22.70 ± 0.83	0.96	0.29	1.22 × 10^5^	−22.30 ± 1.14	0.94
CMCHIN	0.38	6.02 × 10^4^	−22.50 ± 0.78	0.95	0.21	1.99 × 10^5^	−21.80 ± 1.45	0.96
SM	0.18	1.70 × 10^5^	−21.00 ± 1.25	0.92	0.16	3.21 × 10^5^	−21.10 ± 1.61	0.94
SMCH	0.38	6.17 × 10^4^	−22.60 ± 0.75	0.97	0.29	1.60 × 10^5^	−22.40 ± 1.39	0.97
SMCHIN	0.34	6.91 × 10^4^	−22.50 ± 0.82	0.96	0.21	2.18 × 10^5^	−21.90 ± 1.48	0.97
**PCB 155**								
CM	0.15	1.23 × 10^5^	−20.30 ± 1.20	0.92	0.11	9.74 × 10^4^	−19.40 ± 1.00	0.94
CMCH	0.27	8.12 × 10^4^	−22.00 ± 1.11	0.91	0.15	1.33 × 10^5^	−20.70 ± 1.23	0.98
CMCHIN	0.25	6.59 × 10^4^	−21.50 ± 1.19	0.92	0.15	8.91 × 10^4^	−20.10 ± 1.06	0.93
SM	0.17	7.22 × 10^4^	−20.60 ± 0.87	0.95	0.09	9.27 × 10^4^	−19.10 ± 1.02	0.95
SMCH	0.28	1.12 × 10^5^	−22.50 ± 1.29	0.93	0.15	8.00 × 10^4^	−20.60 ± 0.99	0.93
SMCHIN	0.28	4.13 × 10^4^	−21.60 ± 0.77	0.97	0.22	3.60 × 10^4^	−20.80 ± 0.58	0.97
**PCB 157**								
CM	0.12	8.23 × 10^4^	−20.00 ± 1.10	0.95	0.09	8.36 × 10^4^	−19.40 ± 1.05	0.95
CMCH	0.22	1.29 × 10^5^	−21.90 ± 1.16	0.91	0.17	9.41 × 10^4^	−20.90 ± 0.98	0.96
CMCHIN	0.22	6.66 × 10^4^	−21.20 ± 0.85	0.94	0.16	6.34 × 10^4^	−20.40 ± 0.82	0.97
SM	0.15	3.97 × 10^4^	−19.60 ± 0.75	0.94	0.10	7.79 × 10^4^	−19.60 ± 0.98	0.97
SMCH	0.22	9.49 × 10^4^	−21.60 ± 0.96	0.93	0.23	3.42 × 10^4^	−21.10 ± 0.70	0.96
SMCHIN	0.20	5.97 × 10^4^	−21.30 ± 0.80	0.96	0.21	3.21 × 10^4^	−20.70 ± 0.72	0.96

∆G°: Gibbs free energy (kJ/mol); K_d_: binding affinity; Q_max_: binding capacity (mol/kg); r^2^: correlation coefficient. CMCH: Chlorophyll-amended calcium montmorillonite; CMCHIN: chlorophyllin-amended calcium montmorillonite; CM: calcium montmorillonite; SMCH: chlorophyll-amended sodium montmorillonite; SMCHIN: chlorophyllin-amended sodium montmorillonite; SM: sodium montmorillonite.

## Data Availability

The raw data supporting the conclusions of this article will be made available by the authors on request.

## Supplementary Materials

The following supporting information can be downloaded at https://www.mdpi.com/article/10.3390/toxics14070573/s1, Figure S1: Isotherms showing adsorption of PCB 154 onto binding surfaces of clays at pH 2 (A,B) and pH 6 (C,D); Figure S2: Isotherms showing adsorption of PCB 155 onto binding surfaces of clays at pH 2 (A,B) and pH 6 (C,D); Figure S3: Isotherms showing adsorption of PCB 157 onto binding surfaces of clays at pH 2 (A,B) and pH 6 (C,D); Figure S4: Panels (A–F) show a particular snapshot from a simulation of PCB126, PCB153, PCB154, PCB155, PCB157, and PCB77, respectively, in complex with CM, in acidic conditions; Figure S5: Probability maps showing the contribution of PCB atoms to binding with CM or CMCH and their corresponding dihedral angles in acidic conditions; Figure S6: Probability maps showing the contribution of PCB atoms to binding with CM or CMCH and their corresponding dihedral angles, in near-neutral conditions; Figure S7: Panels (A–F) show a particular snapshot from a simulation of PCB126, PCB77, PCB153, PCB154, PCB155, and PCB157, respectively, in complex with CMCH, in acidic conditions; Figure S8: The panels (A,B) show the average contribution of chlorophyll’s chemical groups to the interactions with six PCB molecules (PCB126, PCB153, PCB154, PCB155, PCB157, and PCB77) in acidic and near-neutral conditions, respectively.
