# Supplementary material for: Green-Engineered Clays Tightly Adsorb and Detoxify Environmentally Persistent Polychlorinated Biphenyls and Complex Mixtures
Source: Toxics. 2026 Jun 29;14(7):573. doi: 10.3390/toxics14070573 (PMC13418004; doi:10.3390/toxics14070573)
Supplement: Supplementary file 1 [file toxics-14-00573-s001.zip › toxics-4344333-supplementary.pdf]

Green-Engineered Clays Tightly Adsorb and Detoxify Environmentally Persistent Polychlorinated Biphenyls and Complex Mixtures

Supporting Figures

Johnson O. Oladele [1,2], Xenophon Xenophontos [3], Phanourios Tamamis [3,4], Stephen Safe [1,2] and Timothy D. Phillips [1,2,*]

[1] Interdisciplinary Faculty of Toxicology, Texas A&M University, College Station, TX 77843, USA; oladelejohn2007@tamu.edu (J.O.O.); ssafe@cvm.tamu.edu (S.S.)
[2] Department of Veterinary Physiology and Pharmacology, College of Veterinary Medicine & Biomedical Sciences, Texas A&M University, College Station, TX 77843, USA
[3] Artie McFerrin Department of Chemical Engineering, College of Engineering, Texas A&M University, College Station, TX 77843, USA; xxenop01@tamu.edu (X.X.); tamamis@tamu.edu (P.T.)
[4] Department of Materials Science and Engineering, College of Engineering, Texas A&M University, College Station, TX 77840, USA
* Correspondence: tphillips@cvm.tamu.edu

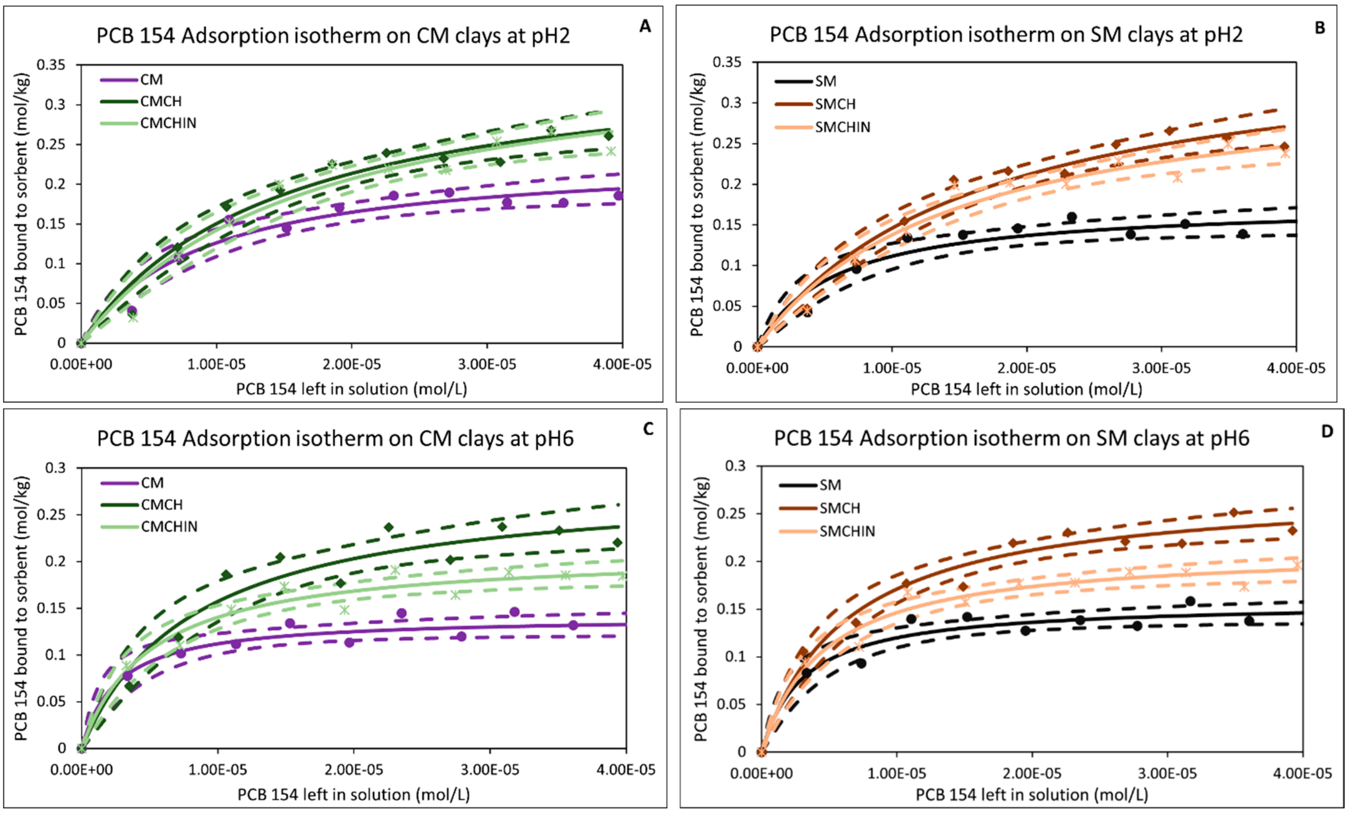

Figure S1. Isotherms showing adsorption of PCB 154 onto binding surfaces of clays at pH 2 (A & B) and pH 6 (C & D). CMCH: chlorophyll-amended calcium montmorillonite; CMCHIN: chlorophyllin-amended calcium montmorillonite; CM: calcium montmorillonite; SMCH: chlorophyll-amended sodium montmorillonite; SMCHIN: chlorophyllin-amended sodium montmorillonite; SM: sodium montmorillonite.

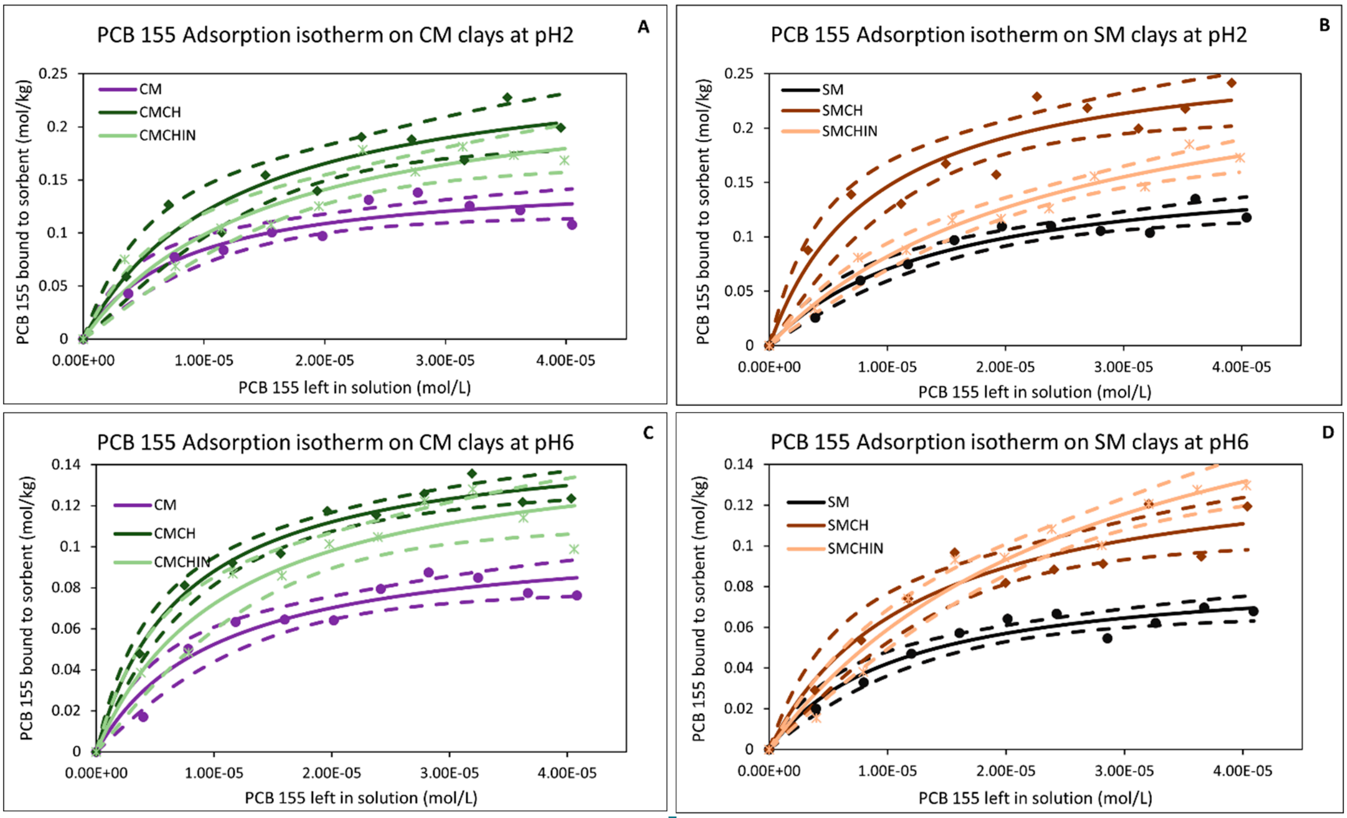

Figure S2. Isotherms showing adsorption of PCB 155 onto binding surfaces of clays at pH 2 (A & B) and pH 6 (C & D).

CMCH: chlorophyll-amended calcium montmorillonite; CMCHIN: chlorophyllin-amended calcium montmorillonite; CM: calcium montmorillonite; SMCH: chlorophyll-amended sodium montmorillonite; SMCHIN: chlorophyllin-amended sodium montmorillonite; SM: sodium montmorillonite.

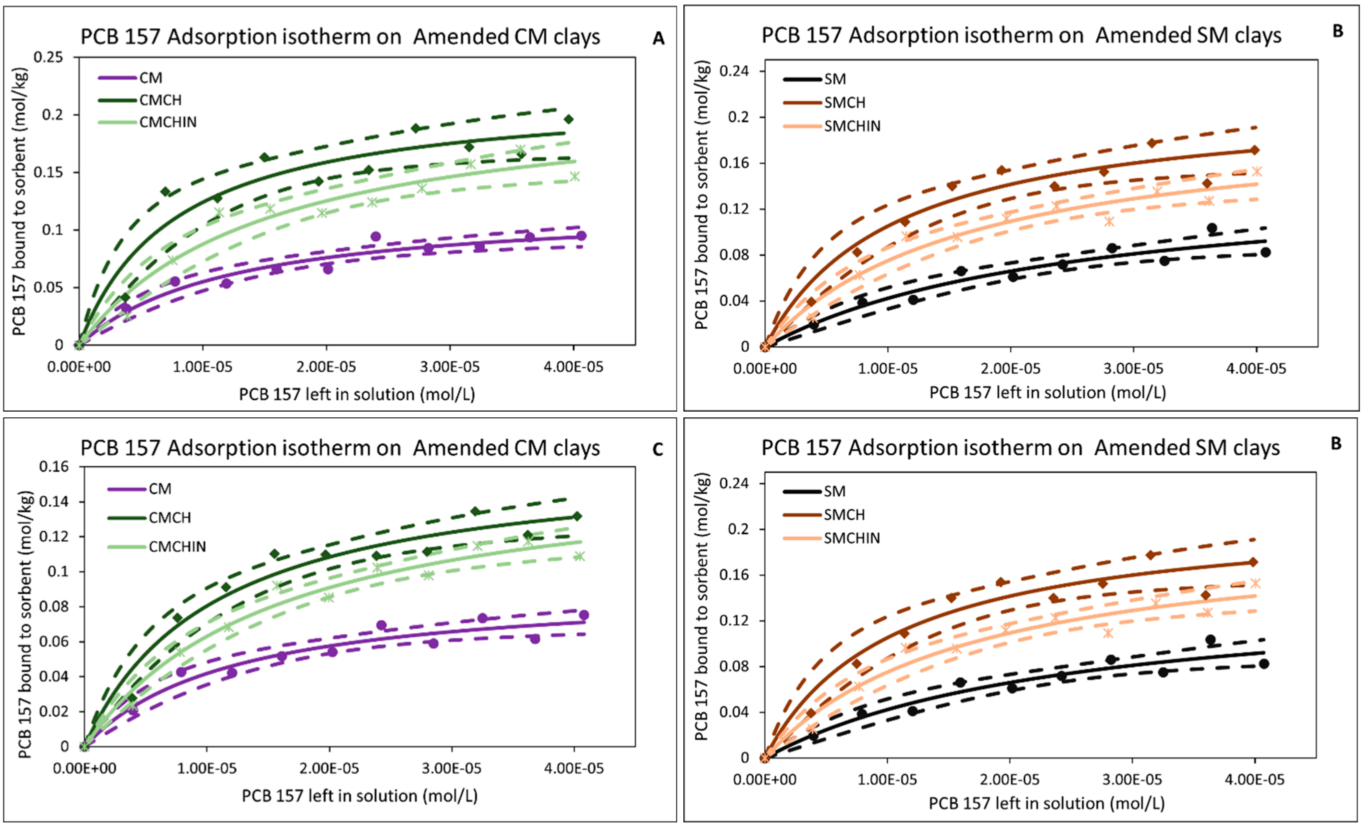

Figure S3. Isotherms showing adsorption of PCB 157 onto binding surfaces of clays at pH 2 (A & B) and pH 6 (C & D).

CMCH: chlorophyll-amended calcium montmorillonite; CMCHIN: chlorophyllin-amended calcium montmorillonite; CM: calcium montmorillonite; SMCH: chlorophyll-amended sodium montmorillonite; SMCHIN: chlorophyllin-amended sodium montmorillonite; SM: sodium montmorillonite.

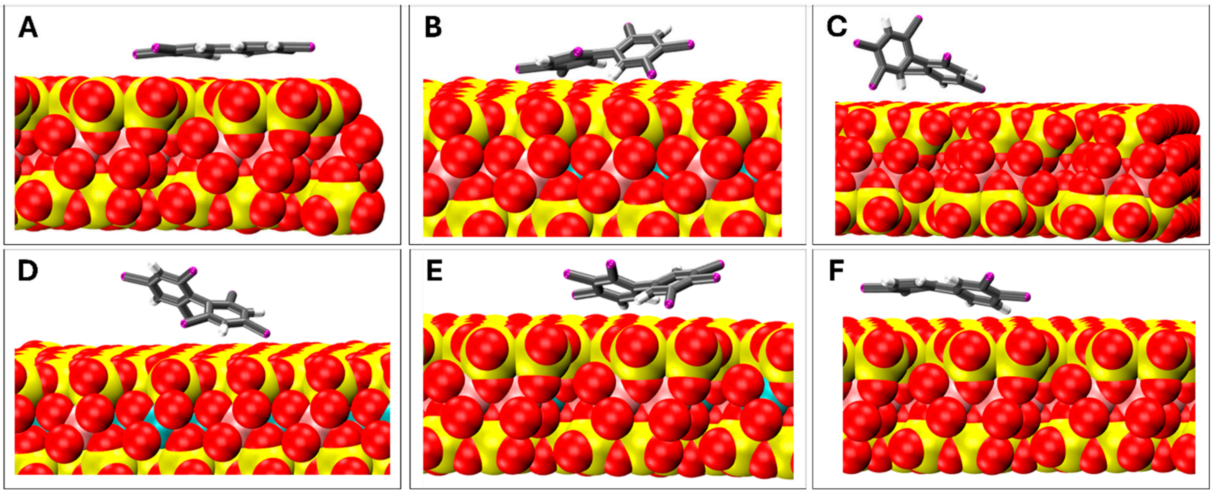

Figure S4: Panels A-F show a particular snapshot from a simulation of PCB126, PCB153, PCB154, PCB155, PCB157, and PCB77, respectively, in complex with CM, in acidic conditions.

The snapshots were selected to show PCB-CM interactions. The clay layers are shown in vdW representation, colored by atom type. The PCB molecules are shown in licorice representation, with the carbon atoms colored in black, the chlorine atoms colored in purple, and the hydrogen atoms colored in white. The hydrogen atoms of clay, as well as the water molecules and ions were omitted for clarity. All representations were produced using VMD.

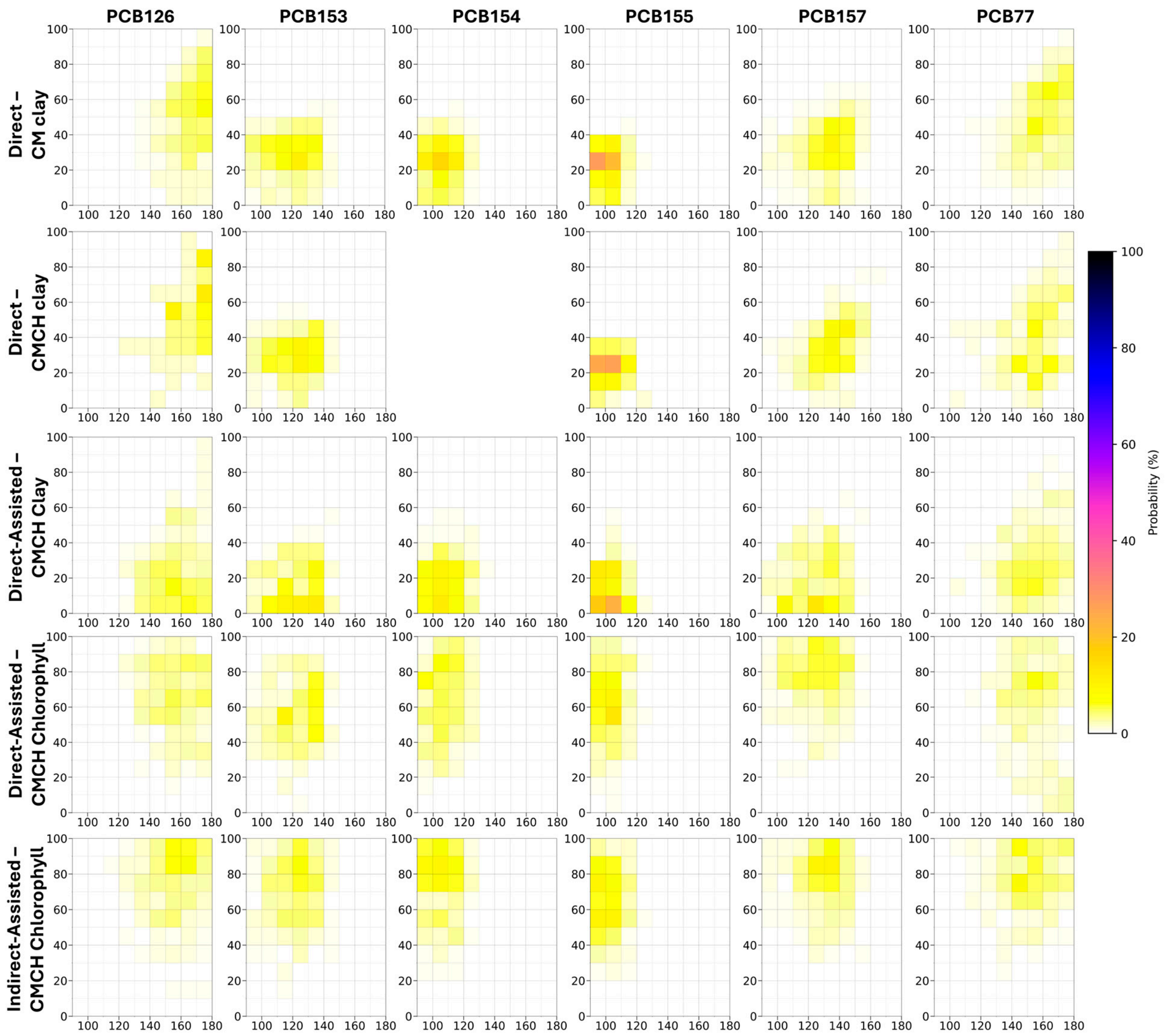

Figure S5: Probability maps showing the contribution of PCB atoms to binding with CM or CMCH and their corresponding dihedral angles in acidic conditions. The y-axes represent the percentage contribution of PCB atoms to binding (0–100%), while the x-axes represent the dihedral angle of PCB molecules (90–180°). Cell colors indicate the probability of a PCB molecule interacting with CM or CMCH at a given atom contribution and dihedral angle. The maps are arranged in six columns and five rows. Columns (left to right) correspond to PCB126, PCB153, PCB154, PCB155, PCB157, and PCB77, respectively. Rows (top to bottom) correspond to (i) direct interactions with the CM clay surface, (ii) direct interactions with the CMCH clay surface, (iii) direct-assisted interactions with the CMCH clay surface, (iv) direct-assisted interactions with CMCH chlorophyll molecules, and (v) indirect-assisted interactions with CMCH chlorophyll molecules. The probability map corresponding to direct interactions of PCB154 with the clay surface of CMCH is not shown, as the direct interactions of PCB154 with the clay surface of CMCH across the five runs were negligible. Values were calculated from the last 50 ns of five independent simulations.

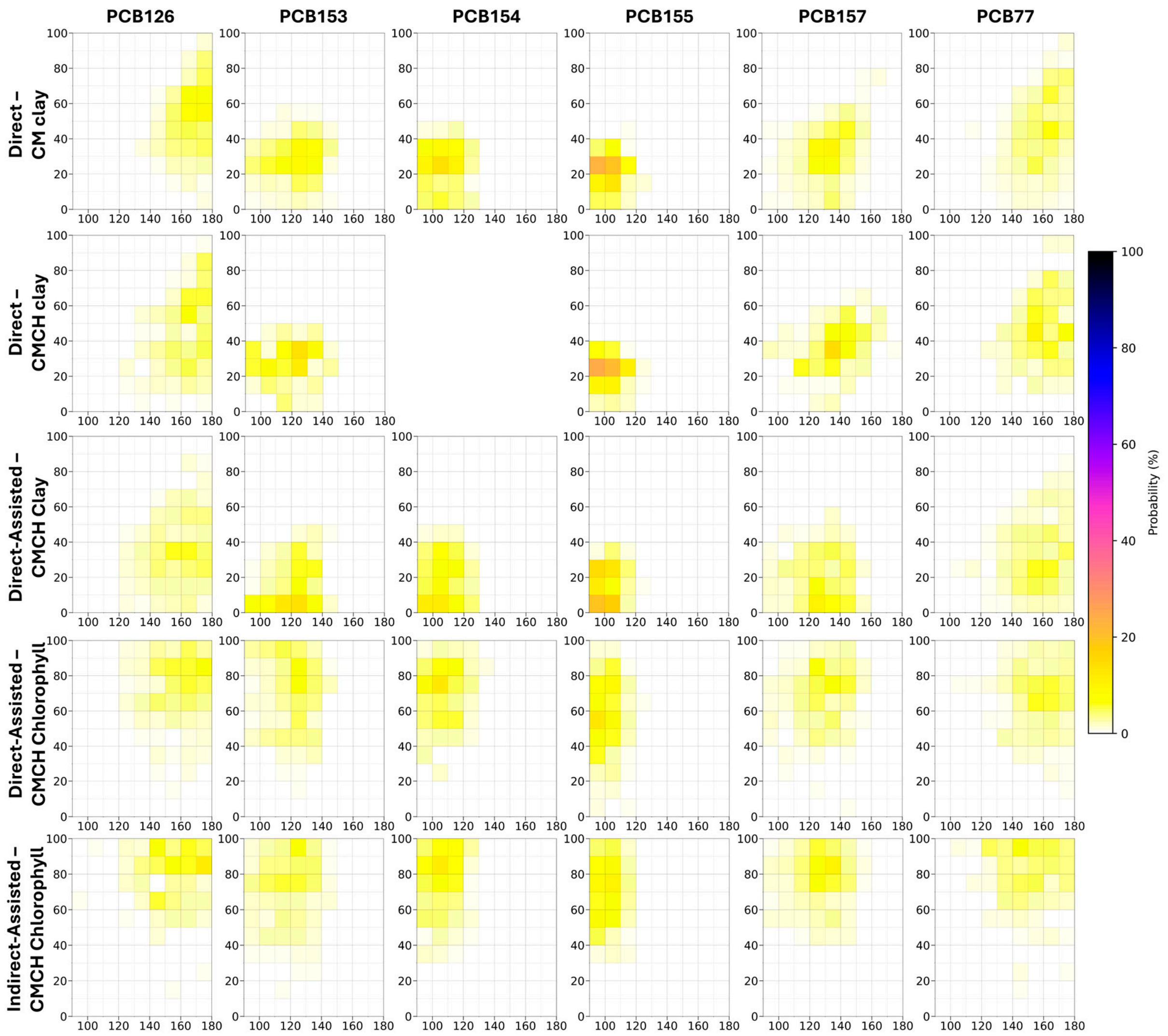

Figure S6: Probability maps showing the contribution of PCB atoms to binding with CM or CMCH and their corresponding dihedral angles, in near-neutral conditions. The y-axes represent the percentage contribution of PCB atoms to binding (0–100%), while the x-axes represent the dihedral angle of PCB molecules (90–180°). Cell colors indicate the probability of a PCB molecule interacting with CM or CMCH at a given atom contribution and dihedral angle. The maps are arranged in six columns and five rows. Columns (left to right) correspond to PCB126, PCB153, PCB154, PCB155, PCB157, and PCB77, respectively. Rows (top to bottom) correspond to (i) direct interactions with the CM clay surface, (ii) direct interactions with the CMCH clay surface, (iii) direct-assisted interactions with the CMCH clay surface, (iv) direct-assisted interactions with CMCH chlorophyll molecules, and (v) indirect-assisted interactions with CMCH chlorophyll molecules. The probability map corresponding to direct interactions of PCB154 with the clay surface of CMCH is not shown, as the direct interactions of PCB154 with the clay surface of CMCH across the five runs were negligible. Values were calculated from the last 50 ns of five independent simulations.

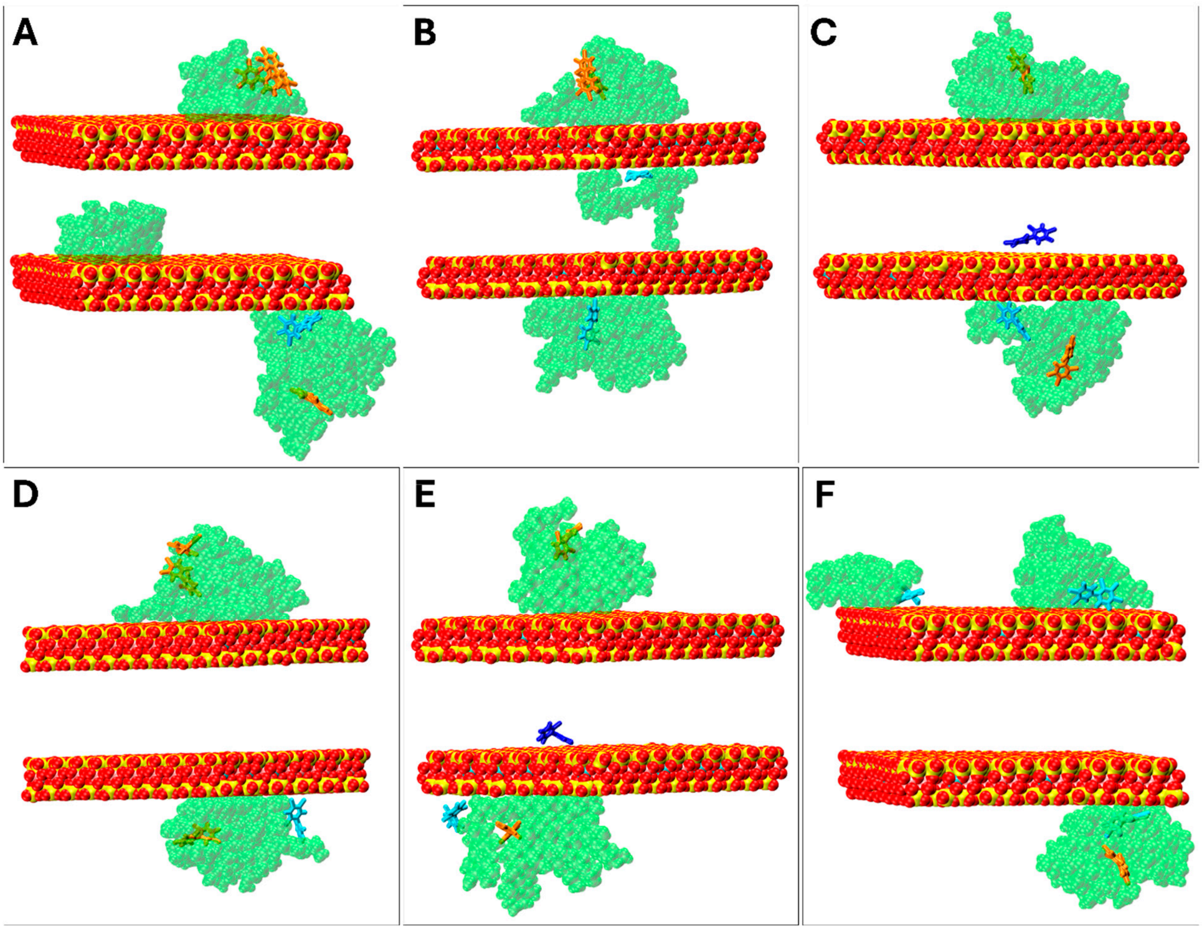

Figure S7: Panels A-F show a particular snapshot from a simulation of PCB126, PCB77, PCB153, PCB154, PCB155, and PCB157, respectively, in complex with CMCH, in acidic conditions. The clay layers are shown in vdW representation, colored by atom type. Chlorophyll molecules are shown in transparent vdW representation, colored in green. The PCB molecules are shown in licorice representation, colored in blue, cyan or orange, corresponding to direct, direct-assisted, and indirect-assisted interactions, respectively. The hydrogen atoms of clay, as well as the water molecules and ions were omitted for clarity. All representations were produced using VMD.

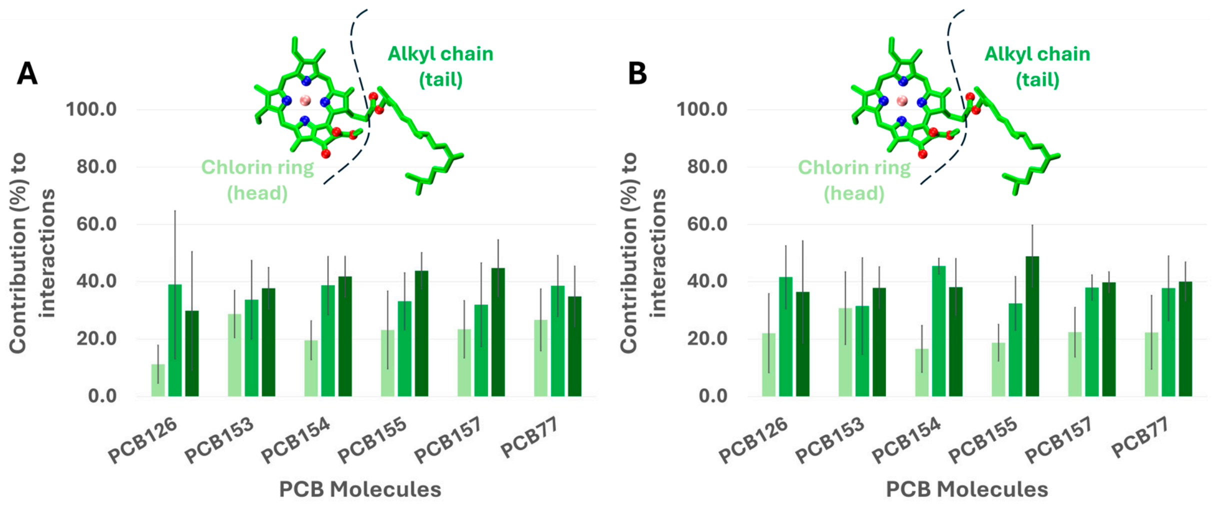

Figure S8: The panels A and B show the average contribution of chlorophyll's chemical groups to the interactions with six PCB molecules (PCB126, PCB153, PCB154, PCB155, PCB157, and PCB77) in acidic and near-neutral conditions, respectively. The average contribution of chlorophyll's "head", "tail", or both moieties to the interactions with the six PCB molecules is shown in light blue, red, and green, respectively. The average values were calculated using the last 50 ns of five independent simulations; error bars denote the standard deviation across simulations.
